# Supplementary material for: Targeted Social Mobilization in a Global Manhunt
Source: PLoS One. 2013 Sep 30;8(9):e74628. doi: 10.1371/journal.pone.0074628 (PMC3786994; doi:10.1371/journal.pone.0074628)
Supplement: File S1 — Supporting information for the manuscript, including Figures S1–S4 and Tables S1–S7. Figure S1, Heat map of raw ight numbers. Figure S2, Heat map of normalised and locality-adjusted adjacency matrix with greediness set to 0.3. Figure S3, Plot of stationary distribution during a random walk on reduced MSA network (comprising only North America and Europe), with increasing degree of greediness moving clockwise from top left. Figure S4, Heatmap showing tra_c to crowdscanner.com on 48 hours approaching the challenge. Table S1, Table of regional hub MSA's and centralities. Table S2, Table of MSA's with highest centrality values after locality adjustment (and tag cities for comparison). Table S3, List of cities in North America. Table S4, List of cities in South America. Table S5, List of cities in Europe. Table S6, List of cities in Asia. Table S7, List of cities in Africa (PDF) [file pone.0074628.s001.pdf]

Supporting online material for:

Targeted Social Mobilization in a Global Manhunt

Contents

A Flight Network 1

B Reduced Network 4

C Website Traffic 5

D List of Cities 5

A Flight Network

We can visualise the adjacency matrix of the MSA network both in terms of raw number of flights (Fig (1)) and the normalised, locality and greediness-adjusted edges (Fig (2)). There are 2 striking features in Figure (1), firstly we see a strong community structure with respect to continents as also observed in [1], particularly within Asia and secondly the high occupation of the diagonal. While the intercontinent connectivity is intuitively understandable, the latter is explained by geography. In regions such as Polynesia, there are a large number of flights between small regional airports on different islands but few outside of the community. Among the MSA's which represented the largest number of airports, were Jakarta (Indonesia), Auckland (New Zealand), Anchorage (Alaska, USA) and Port Moresby (Papua New Guinea) which are all regional hubs within sparsely-populated or archipelagic areas which may only feasibly be navigated by air. Since these small regional airports all agglomerate to a single MSA, it appears that a large volume of flights appear to leave from and arrive at the same MSA. Therefore these hubs have large unadjusted localities represented by large values along the diagonal. This artifact of the agglomeration process has a negligble affect on the structure of the network as a whole since these communities are not particularly central; this can be seen by the low centralities of these MSA's

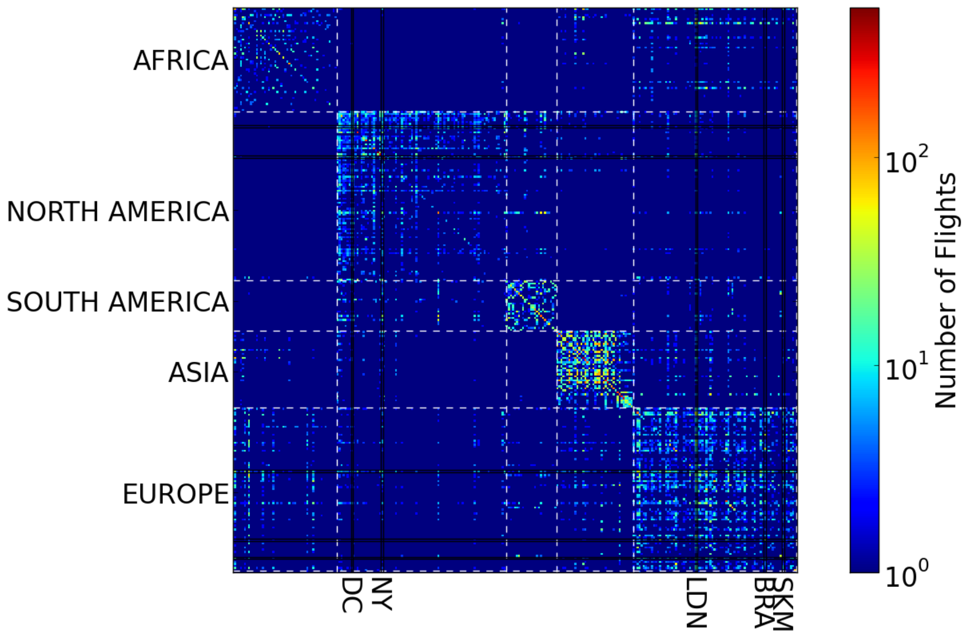

Figure 1: Heat map of raw flight numbers. Continent limits are marked by white dashed lines and tag cities with black lines.

Table 1: Table of regional hub MSA's and centralities

MSA	Centrality	Centrality/Centrality$_{equal}$
Jakarta	0.00572	1.24
Auckland	0.00289	0.63
Anchorage	0.00092	0.20
Port Moresby	0.00176	0.38
Equal	0.0046	

The adjusted adjacency matrix used in the simulations and shown in Figure (2) maintains the strong continental community effect, however the localities have been uniformly set to 0.39 and a greediness of 30% has been applied. In a few cases this greediness leads to increased locality if all outgoing edges from an MSA move the message away from the nearest Tag city. The greediness also leads to a number of strong connections to Europe (but not directly the the European Tag cities); the turquoise dots representing strength 0.3 in the columns on the right of the figure.

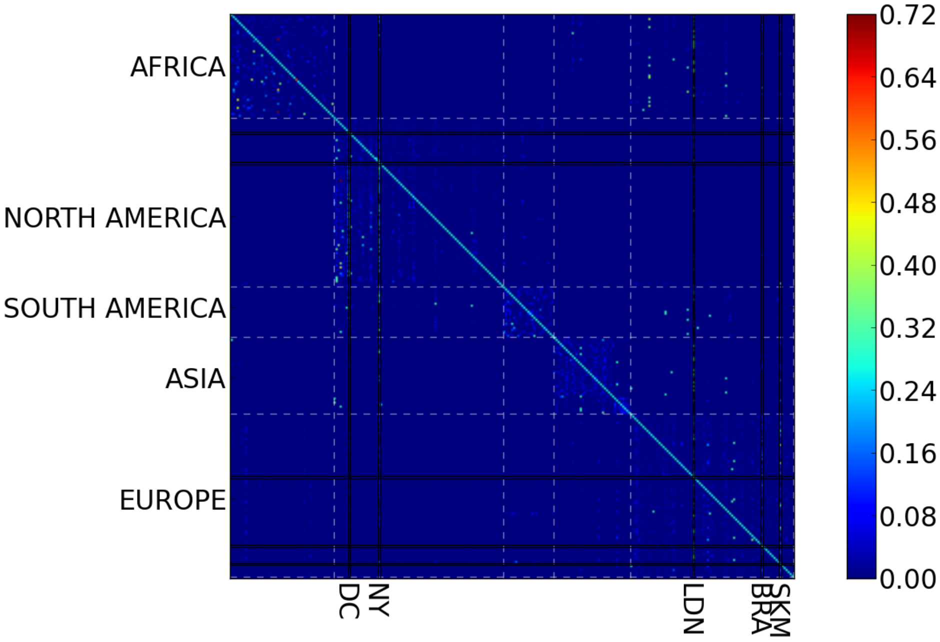

Figure 2: Heat map of normalised and locality-adjusted adjacency matrix with greediness set to 0.3. Continent limits are marked by white dashed lines and tag cities with black lines.

Table (2) shows the centralities of the most central MSAs in the network along with the Tag cities for comparison. All of the tag cities are above the baseline of equal centrality amongst all the nodes, however London, Washington DC and NY are expecially so.

Table 2: Table of MSA's with highest centrality values after locality adjustment (and tag cities for comparison)

MSA	Centrality	Centrality/Centrality$_{\text{equal}}$
Shanghai	0.02242	6.28
London	0.02231	6.25
Chongquing	0.01988	5.57
Beijing	0.01745	4.89
Barcelona	0.0155	4.34
Milan	0.0149	4.17
London	0.02225	6.25
NY	0.010	2.80
DC	0.00645	1.87
Bratislava/Vienna	0.0054	1.51
Stockholm	0.0059	1.65
Equal	0.00357	

B Reduced Network

Figure (3) shows the effective centralities of the cities within a reduced network comprising the cities of North America and Europe only (compare with the full global network shown in Fig (6) in the main paper). A degree of targeting of 30% now leads to a proportion of messages reaching the tag cities of 0.50 (compared to 0.44 using the full network). As expected the proportion of time spent in the tag cities increases as nodes are removed from the network. In fact the effect of the removal of the South American, African and Asian MSA's becomes smaller as targeting becomes stronger and routes the message towards the western hemisphere. Considering the pure, non-targeting random walk the reduced network increases the Tag proportion from 0.05 to 0.09; a percentage increase of 80%. However as the targeting becomes stronger this percentage difference becomes smaller. When greediness is set to 30% the reduced network increases the tag proportion from 0.44 to 0.50, an increase of only 13%.

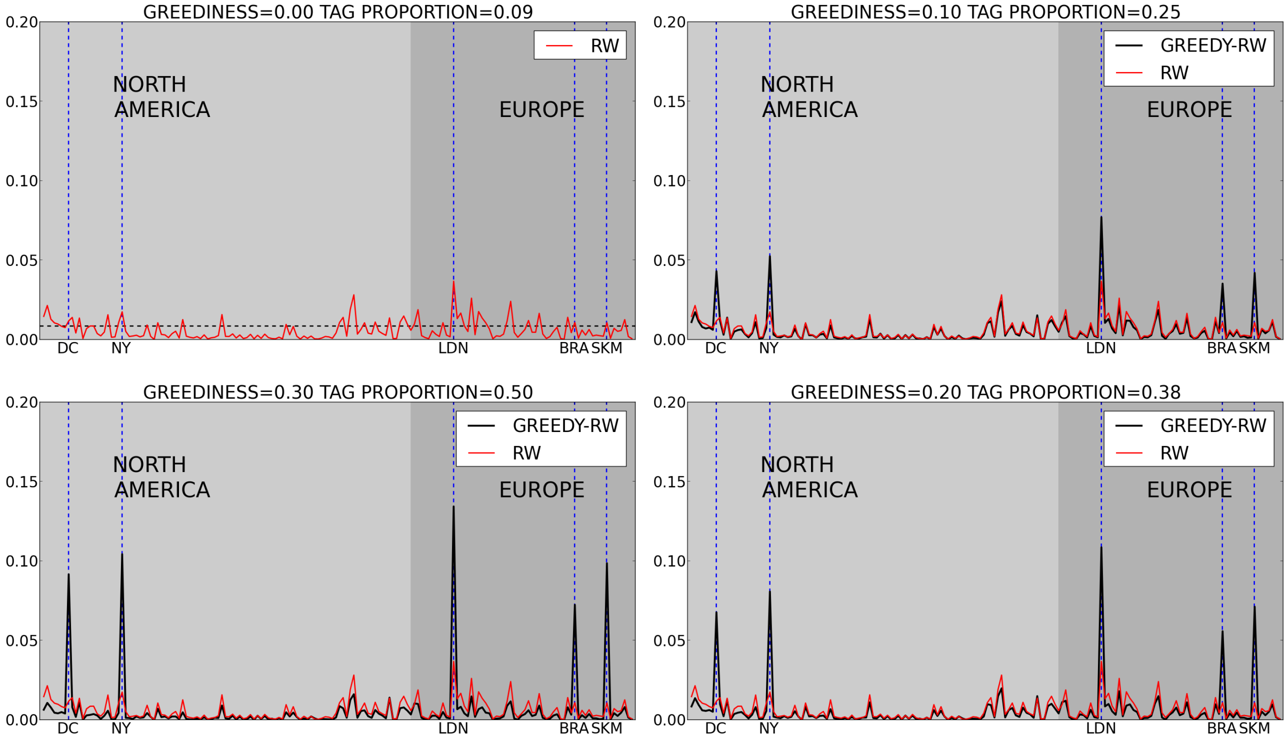

Figure 3: Plot of stationary distribution during a random walk on reduced MSA network (comprising only North America and Europe), with increasing degree of greediness moving clockwise from top left. The red line represents an unbiased random walk, corresponding to pure random mobilization via broadcast messaging. (Top left) The horizontal dashed line represents the uniform distribution of centralities expected in a fully connected graph. The black line in other plots represents a greedy random walk.

C Website Traffic

Figure (4) shows the geographical distribution of traffic to our team's website in the 48 hours approaching the challenge. Traffic overwhelmingly originates from Europe and North America, particularly since this snapshot is from the critical latter stages of the propagation process, but we can also notice the presence of traffic originating from South America, Australia and Asia Pacific. The fact that tag traffic is significant even outside the Anglosphere suggests that the information diffusion either took place in languages other than English (a small but significant number of tweets were in languages other than English) or the English language media exposure was accessible via the *lingua franca*. While the South American, Asian and African nodes clearly participated in the diffusion, the network upon which this took place is likely somewhere between the reduced network presented here and the full global network presented in the main paper. Regardless of which extreme of network substrate dominates, we can conclude that significant targeting is required to reproduce the proportions of traffic towards the Tag cities.

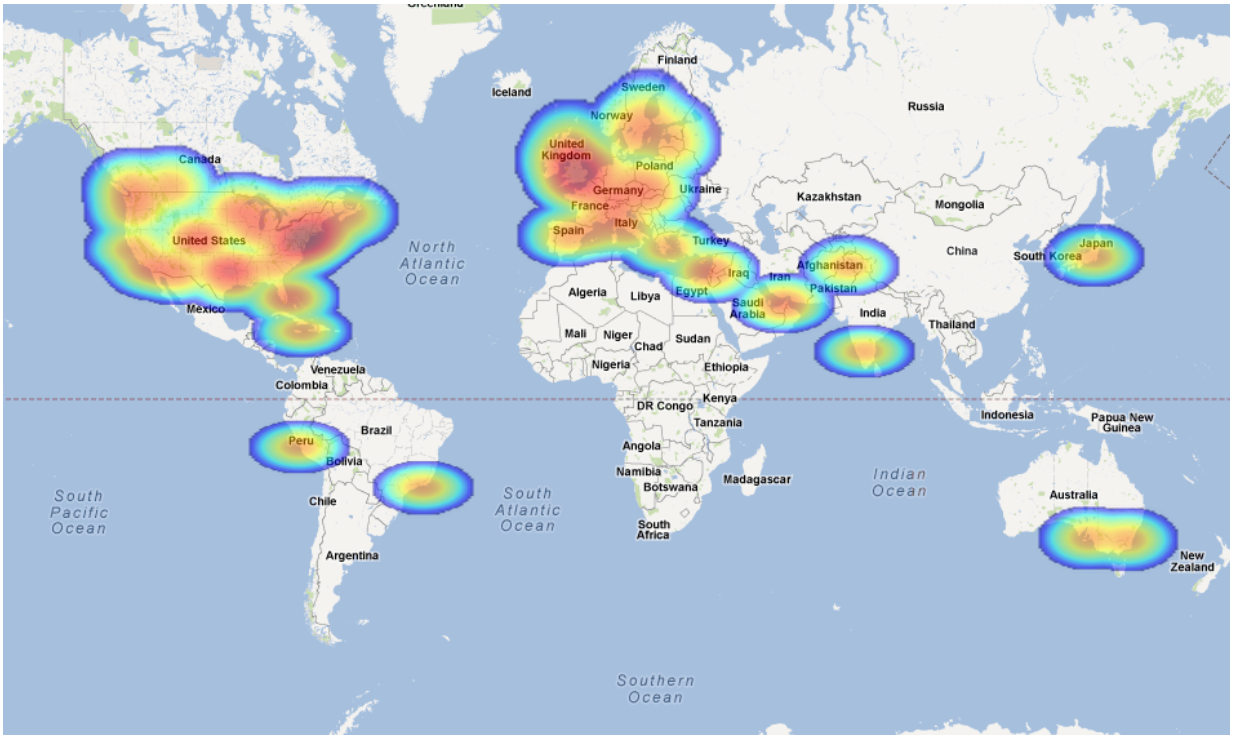

Figure 4: Heatmap showing traffic to crowdscanner.com on 48 hours approaching the challenge.

D List of Cities

References

[1] Guimera R, Mossa S, Turtschi A, Amaral LAN (2005) The worldwide air transportation network: Anomalous centrality, community structure, and cities' global roles. *Proceedings of the National Academy of Sciences* 102:7794–7799.

Table 3: List of cities in North America

Los Angeles	Chicago
Toronto	Dallas
Houston	Philadelphia
Detroit	Washington
Atlanta	San Diego
Boston	Riverside
Phoenix	Seattle
Minneapolis	Tampa
Saint Louis	Baltimore
Denver	El Paso
Anchorage	Vancouver
New York	Honolulu
San Antonio	San Jose
Jacksonville	Indianapolis
Austin	San Francisco
Columbus	Fort Worth
Charlotte	Detroit
El Paso	Memphis
Boston	Nashville
Denver	Baltimore
Louisville	Milwaukee
Portland	Oklahoma City
Las Vegas	Albuquerque
Tucson	Fresno
Sacramento	Long Beach
Kansas City	Virginia Beach
Corpus Christi	Colorado Springs
Raleigh	Omaha
Miami	Tulsa
Oakland	Cleveland
Minneapolis	Wichita
New Orleans	Bakersfield
Tampa	Honolulu
Santa Ana	St. Louis
Pittsburgh	Lexington
Cincinnati	Anchorage
Saint Paul	Greensboro
Lincoln	Buffalo
Fort Wayne	Saint Petersburg
Orlando	Norfolk
Laredo	Madison
Lubbock	Durham
WinstonSalem	Garland
Baton Rouge	Reno
Scottsdale	San Bernardino
Birmingham	Rochester

Table 4: List of cities in South America

City	Country
Mexico City	Mexico
Sâo Paulo	Brazil
Buenos Aires	Argentina
Rio de Janeiro	Brazil
Lima	Peru
Bogotá	Colombia
Caracas	Venezuala
Santiago	Chile
Belo Horizonte	Brazil
Guadalajara	Mexico
Monterrey	Mexico
Porto Alegre	Brazil
Recife	Brazil
Salvador	Brazil
Brasilia	Brazil
Medelln	Colombia
Fortaleza	Brazil
Santo Domingo	Dominic Republic
Curitiba	Brazil
Guatemala City	Guatemala
Cali	Colombia
Guayaquil	Ecuador
Puebla	Mexico
Campinas	Brazil
San Juan	Puerto Rico

Table 5: List of cities in Europe

City	Country	City	Country
Amsterdam	Netherlands	Antwerp	Belgium
Athens	Greece	Barcelona	Spain
Baku	Azerbaijan	Belgrade	Serbia
Berlin	Germany	Bremen	Germany
Bristol	United Kingdom	Brussels	Belgium
Bucharest	Romania	Budapest	Hungary
Cardiff	United Kingdom	Copenhagen	Denmark
Donetsk	Ukraine	Dnipropetrovsk	Ukraine
Dublin	Ireland	Frankfurt	Germany
Glasgow	United Kingdom	Hamburg	Germany
Helsinki	Finland	Istanbul	Turkey
Katowice	Poland	Kazan	Russia
Kharkiv	Ukraine	Kiev	Ukraine
Krakow	Poland	Leeds	United Kingdom
Lisbon	Portugal	Liverpool	United Kingdom
Łódź	Poland	London	United Kingdom
Lyon	France	Madrid	Spain
Manchester	United Kingdom	Marseille	France
Milan	Italy	Minsk	Belarus
Moscow	Russia	Munich	Germany
Napoli	Italy	Nice	France
Nizhniy Novgorod	Russia	Nottingham	United Kingdom
Nuremberg	Germany	Odessa	Ukraine
Oslo	Norway	Paris	France
Perm	Russia	Porto	Portugal
Portsmouth	United Kingdom	Prague	Czech Republic
Düsseldorf	Germany	Cologne	Germany
Riga	Latvia	Roma	Italy
Rostov-on-Don	Russia	Rotterdam	Netherlands
Dortmund	Germany	Saarbrücken	Germany
Saint Petersburg	Russia	Samara	Russia
Saratov	Russia	Seville	Spain
Sofia	Bulgaria	Stockholm	Sweden
Sheffield	United Kingdom	Stuttgart	Germany
Tbilisi	Georgia	Thessaloniki	Greece
Turin	Italy	Newcastle	United Kingdom
Ufa	Russia	Valencia	Spain
Vienna	Austria	Volgograd	Russia
Warsaw	Poland	Birmingham	United Kingdom
Yerevan	Armenia	Zagreb	Croatia
Zurich	Switzerland	Reykjavk	Iceland
Milan	Italy	Katowice	Poland
Lille	France		

Table 6: List of cities in Asia

City	Country	City	Country
Tokyo	Japan	Jakarta	Indonesia
Seoul	South Korea	Delhi	India
Mumbai	India	Manila	Philippines
Shanghai	China	Osaka	Japan
Kolkata	India	Karachi	Pakistan
Guangzhou	China	Dhaka	Bangladesh
Shenzhen	China	Tehran	Iran
Beijing	China	Bangkok	Thailand
Chennai	India	Nagoya	Japan
Bangalore	India	Hong Kong	Hong Kong
Wuhan	China	Taipei	Taiwan
Lahore	Pakistan	Tianjin	China
Kuala Lumpur	Malysia	Chongqing	China
Hyderabad	India	Ho Chi Min City	Vietnam
Shenyang	China	Ahmedabad	India
Brisbane	Australia	Samarkand	Uzbekistan
Auckland	New Zealan	Port Moresby	Papua New Guineau
Sydney	Australia	Melbourne	Australia
Perth	Australia	Adelaide	Australia

Table 7: List of cities in Africa

City	Country
Cairo	Egypt
Lagos	Nigeria
Kinshasa	Democratic Republic of the Congo
Johannesburg	South Africa
Khartoum	Sudan
Alexandria	Egypt
Abidjan	Ivory Coast
Casablanca	Morocco
Cape Town	South Africa
Durban	South Africa
Accra	Ghana
Nairobi	Kenya
Ibadan	Nigeria
Dar es Salaam	Tanzania
Algiers	Algeria
Addis Ababa	Ethiopia
Luanda	Angola
Dakar	Senegal
Pretoria	South Africa
Tripoli	Libya
Harare	Zimbabwe
Douala	Cameroon
Hargeisa	Republic of Somaliland and Somalia
Abuja	Nigeria
Kampala	Uganda
Bamako	Mali
Maputo	Mozambique
Antananarivo	Madagascar
Lusaka	Zambia
Yaounde	Cameroon
Ouagadougou	Burkina Faso
Conakry	Guinea
Kumasi	Ghana
Lubumbashi	Democratic Republic of the Congo
Mbuji	Democratic Republic of the Congo
Brazzaville	Republic of the Congo
Oran	Algeria
Benin	Nigeria
Port Harcourt	Nigeria
Tunis	Tunisia
Freetown	Sierra Leone
Cotonou	Benin
Vereeniging	South Africa
Fes	Morocco
Maiduguri	Nigeria
Monrovia	Liberia
Port Elizabeth	South Africa
Huambo	Angola
Ogbomosho	Nigeria
Zaria	Nigeria
Ndjamena	Chad
